# Efficacy of Tricaine (MS-222) and Hypothermia as Anesthetic Agents for Blocking Sensorimotor Responses in Larval Zebrafish

**DOI:** 10.3389/fvets.2022.864573

**Published:** 2022-03-28

**Authors:** Claire Leyden, Timo Brüggemann, Florentyna Debinski, Clara A. Simacek, Florian A. Dehmelt, Aristides B. Arrenberg

**Affiliations:** ^1^Werner Reichardt Centre for Integrative Neuroscience and Institute for Neurobiology, University of Tuebingen, Tuebingen, Germany; ^2^Graduate Training Centre of Neuroscience, University of Tuebingen, Tuebingen, Germany

**Keywords:** zebrafish, animal welfare, 3R, tricaine, gradual cooling, anesthesia, MS-222, optokinetic response

## Abstract

Tricaine, or MS-222, is the most commonly used chemical anesthetic in zebrafish research. It is thought to act via blocking voltage-gated sodium channels, though its mechanism of action, particularly at the neuronal level, is not yet fully understood. Here, we first characterized the effects of tricaine on both body balance and touch responses in freely swimming animals, before determining its effect on the neural activity underlying the optokinetic response at the level of motion perception, sensorimotor signaling and the generation of behavior in immobilized animals. We found that the standard dose for larvae (168 mg/L) induced loss of righting reflex within 30 seconds, which then recovered within 3 minutes. Optokinetic behavior recovered within 15 minutes. Calcium imaging showed that tricaine interferes with optokinetic behavior by interruption of the signals between the pretectum and hindbrain. The motion sensitivity indices of identified sensory neurons were unchanged in larvae exposed to tricaine, though fewer such neurons were detected, leaving a small population of active sensory neurons. We then compared tricaine with gradual cooling, a potential non-chemical alternative method of anesthesia. While neuronal tuning appeared to be affected in a similar manner during gradual cooling, gradual cooling induced a surge in calcium levels in both the pretectum and hindbrain. This calcium surge, alongside a drop in heartrate, is potentially associated with harmful changes in physiology and suggests that tricaine is a better anesthetic agent than gradual cooling for zebrafish laboratory research.

## Introduction

Zebrafish are one of the most commonly used model organisms in biological research; it was suggested in 2017 that more than 5 million zebrafish were used annually (1), and that number has only continued to grow. Understanding animal welfare is crucial to the ethical foundations of such animal experiments, and much work has been devoted to further improve their planning, conduct, reporting and assessment (2–6). For decades, the 3R Principle of animal research (replace, reduce, refine), first introduced by Russell and Burch (7) has formed the core of such efforts, and to this day informs legal regulations of animal research. While several definitions of refinement exist (8, 9), it generally refers to reducing the harmfulness of procedures thus minimizing the suffering of individual animals (4). This includes, but is not limited to, pain (8). While pain and methods to avoid or relieve it are well-understood in some species, they are not in others, including aquatic species (10, 11). Nonetheless, precautions have long been taken to putatively reduce suffering, and one of the most frequent treatments of larval zebrafish is the application of anesthetic agents in preparation for invasive procedures or as a method of euthanasia. Currently, tricaine is the most commonly used laboratory anesthetic, used by 80% of research labs responding to a survey carried out by Lidster et al. (1); despite this fact there has been little investigation into how tricaine acts at the neuronal level in zebrafish. It has been assumed that tricaine preferentially blocks neural signaling in the brain (12), as has been shown in *Xenopus laevis* (13), however, there is a dearth of evidence confirming this claim in zebrafish.

Additionally, multiple studies have found tricaine to be aversive in adult zebrafish, with zebrafish tending to avoid areas where tricaine is present (14, 15). In one of these studies, zebrafish changed their preference in a light/dark box paradigm, from the preferred light side to the non-preferred dark side when tricaine was added (15), showing that this aversion is strong enough to override innate behaviors. It has been noted however, that aversion does not always equate with nociception (16), and the level of animal suffering associated with tricaine administration is thus still unclear.

Any agent that produces a complete or partial loss of feeling can be considered to be an anesthetic, including non-pharmacological agents. Exposure to cold temperatures has recently been observed to have an anesthetic effect in larval zebrafish (17). Gradual cooling has therefore been proposed as an alternative, non-pharmacological anesthetic in zebrafish, but there are comparatively few studies discussing its use, most of which were carried out in adults (18, 19). Similar to the case with tricaine, both of these studies tested the efficacy of gradual cooling at a behavioral level only, finding it to be an effective method of anesthesia. Collymore et al. (18) found that neither tricaine nor gradual cooling led to signs of distress when observing both induction and recovery in adult zebrafish. Behavioral responses during cold treatment are modulated by exposure to analgesics (17), suggesting that nociception could play a role in the overall behavioral reduction observed in cold exposed larvae in the absence of analgesics. Though brain activity in zebrafish is thought to be reduced at low temperatures (20), neural recordings confirming this assumption and characterizing brain responses during anesthetic treatment are missing.

Here, we characterized behavioral, physiological, and neural responses during tricaine treatment and gradual cooling in order to evaluate the possible use of gradual cooling as a method of anesthesia. We assessed the righting reflex and touch responses in freely swimming animals, as well as the neural activity underlying the optokinetic response (OKR) and resultant eye movements in immobilized animals. The OKR consists of reflexive eye movements, the generation of which depends on motion-processing neurons in the visual pretectum and oculomotor neurons in the hindbrain. The investigation of pretectal and hindbrain responses allowed for a direct comparison of sensory and premotor responses to identify at what level along the sensorimotor pathway the necessary activity patterns are lost. We found that tricaine exposure led to a loss of the righting reflex as well as a suppression of reflexive eye movements, though it had only a marginal effect on heartrate. At the neuronal level, tricaine exposure reduced the number of stimulus-associated neurons detected in both the pretectum and hindbrain. While the reduced subset of pretectal neurons exhibited the same tuning as found before treatment, oculomotor-related oscillating hindbrain activity was virtually absent. Gradual cooling, in comparison, had profound effects on evoked eye movements and heartrate, and most critically, induced a surge in calcium levels in both the pretectum and hindbrain. Such calcium waves have previously been associated with apoptosis in larval zebrafish (21). Thus, these results suggest that while gradual cooling may induce a comparable level of anesthesia to tricaine, it has the potential to be more detrimental to the overall health of the animal and the recovery period may, therefore, be inherently more stressful.

## Materials and Methods

### Animals

Animal experiments were performed in accordance with licenses granted by local government authorities (Regierungspräsidium Tübingen) in accordance with German federal law and Baden-Württemberg state law. Approval of this license followed consultation of both in-house animal welfare officers and an external ethics board appointed by the local government. 5–7 days post fertilization (dpf) heterozygous Tg (*elavl3:nls-GCaMP6s)mpn400* zebrafish larvae were used (22). All zebrafish used were also homozygous for the *mitfa* mutation (23). Zebrafish were reared at 29°C on a 14/10 light/dark cycle. Larvae were raised in standard E3 medium containing methylene blue (10^−5^ % v/v) until 3 dpf, when they were sorted for transgene expression and transferred to E3 medium devoid of methylene blue.

### Anesthetics

In order to carry out experiments using tricaine, veterinary-grade tricaine was purchased from PharmaQ (Tricaine Pharmaq 1,000 mg/g). Tricaine was prepared at a concentration of 4 g/L in E3 medium (which did not contain methylene blue) and buffered using 1M Tris (pH 9; 4% v/v) to pH7.

During setup development we found that freezing of the E3 media in the petri-dish may be a problem due to the small volumes of liquid used. To prevent freezing, 1% v/v 1,2-propanediol was added to the E3 medium used in these experiments. Though this only led to minimal reductions in the freezing point (<1°C), this (together with setup improvements) was sufficient and no freezing was seen in any experiments. Control experiments were also carried out at this concentration (referred to as vehicle control throughout). This level has been shown not to be toxic to larval zebrafish (24, 25).

Exposure to either tricaine or gradual cooling had a duration of 7 min, and larvae were exposed only once to a single anesthetic agent. All experiments involving embedded fish included a 6 min baseline period immediately before application of anesthesia.

### Tactile Tests

**Figure 1:**
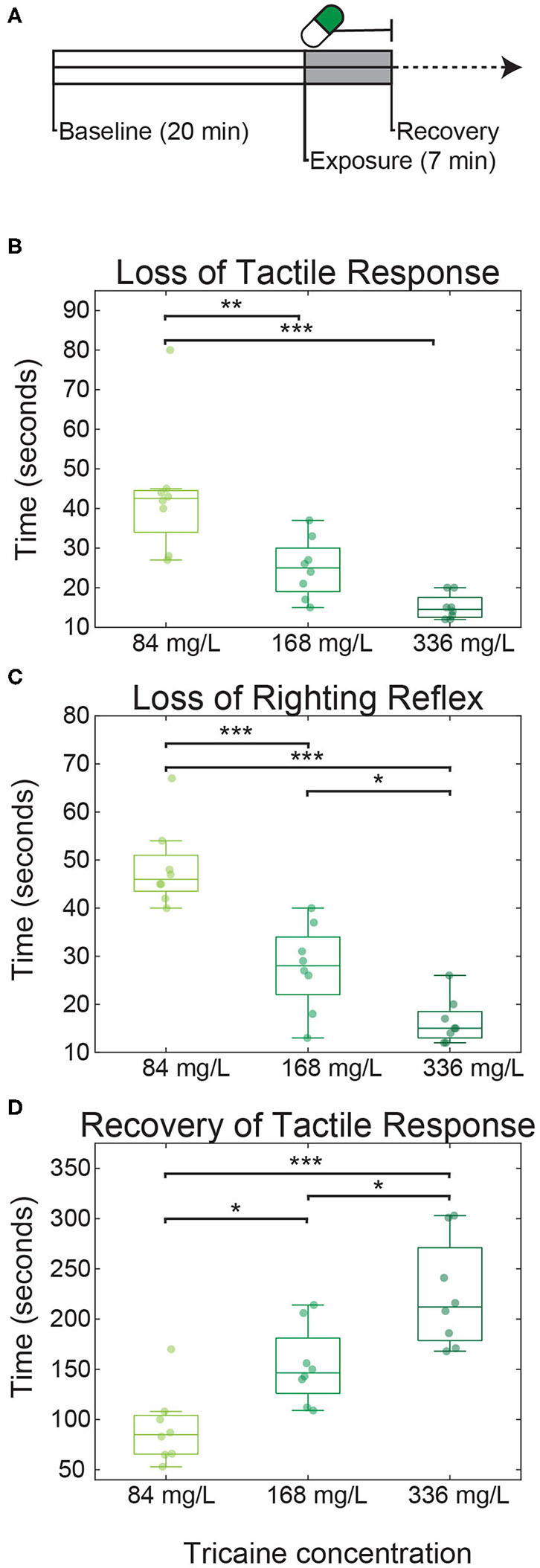
Tricaine onset and recovery in non-restrained zebrafish larvae. **(A)** Larvae were placed in dishes for 20 min to acclimatize (referred to as baseline), followed by 7 min of tricaine exposure. After these 7 min, larvae were transferred to a dish containing tricaine-free E3 and recovery time was assessed. Time to loss of tactile response **(B)**, loss of righting reflex **(C)**, and recovery **(D)** of tactile response were measured in larvae exposed to three concentrations of tricaine: the standard dose (168 mg/L), half this concentration, and double this concentration. Tricaine onset and recovery was dose-dependent, with lower concentrations having the slower onset and faster recovery times. Box plots show quartiles; whiskers extend to the most extreme data points not considered outliers (within 2.7 standard deviations). Results were analyzed via one-way ANOVA with Tukey's HSD test. *N* = 8 for all three conditions, *p < 0.05, **p < 0.005, ***p < 0.001.

Thirty five millimeter petri dishes were filled with 4 mL of E3, then either 168 μL (for control, 0.5X or 1X tricaine conditions) or 336 μL (for 2X tricaine) was removed from the dish, depending on the condition being tested. Individual larvae were placed in dishes and allowed to habituate for 20 min (see Figure 1A). After this habituation period, tricaine was added to the dish (bringing the volume back to 4 mL), and the time required for larvae to lose their righting reflex and cease responding to a tactile stimulus was recorded. The tactile stimulus used was a tap to the tail using a mounting needle. After seven min of tricaine exposure, the fish was transferred to a fresh petri dish containing 4 mL E3, as a first wash step, and then to a second dish, again containing 4 mL E3. The time taken for tactile response to occur was recorded. Data was analyzed via one-way ANOVA with Tukey's honest significant difference (HSD) test, *n* = 8 for all conditions.

### Behavioral OKR Testing

**Figure 2:**
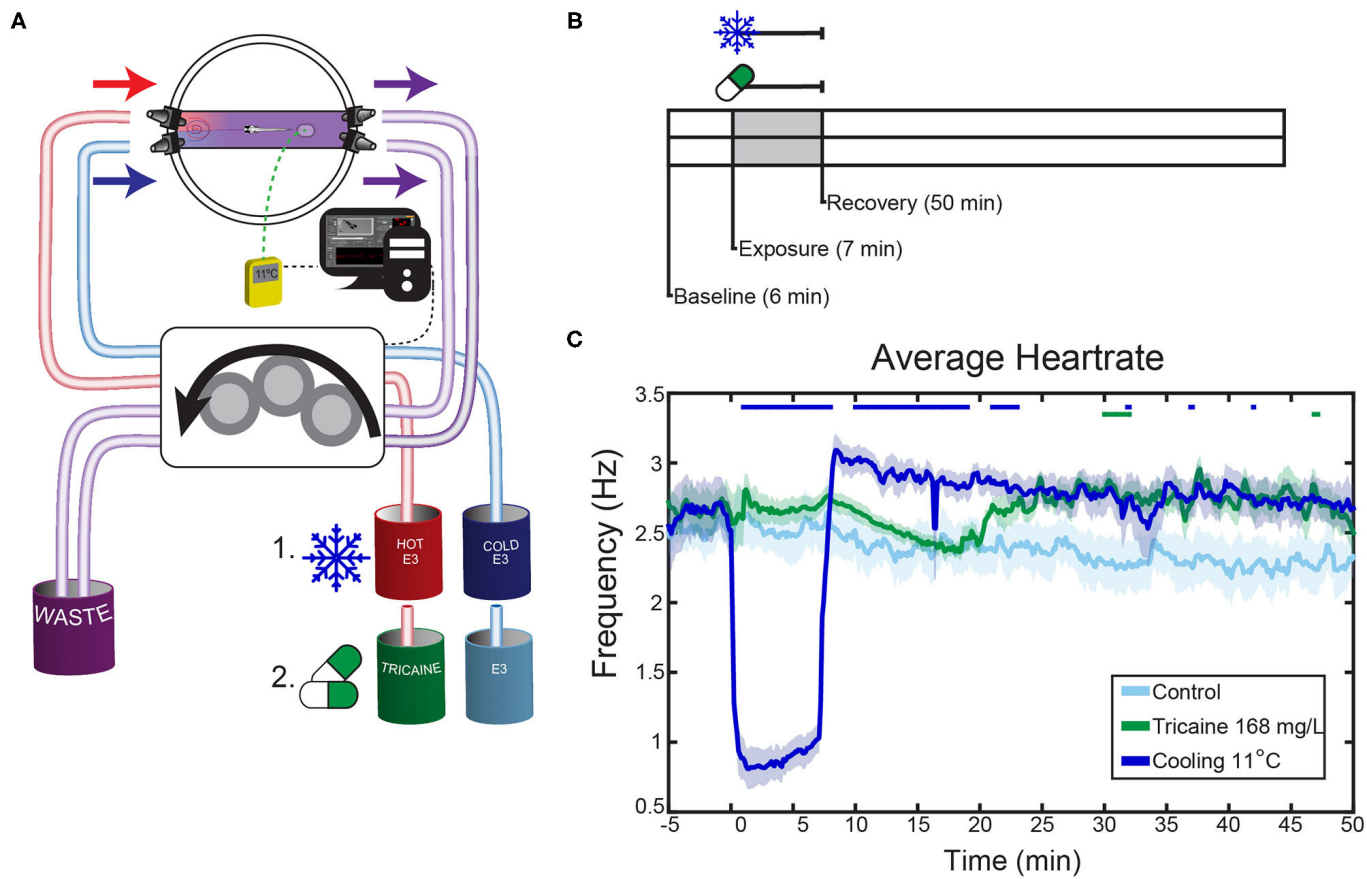
Effects of tricaine and gradual cooling on the heartrate. **(A)** Schematic overview of the experimental setup. Fish were agarose-mounted in glass-bottom petri dishes which were modified to allow liquid to flow through only a narrow channel. The dishes had two connectors on each side in order to apply the liquids, and a sticker to ensure consistent placement of the thermal probe. During gradual cooling experiments, hot and cold E3 were dynamically applied via a peristaltic pump into a dish, where they mixed to achieve the desired temperature under control of a temperature feedback loop (see Methods). In tricaine experiments, the peristaltic pump applied tricaine at the standard concentration (168 mg/L), which was then washed out with drug-free E3. Hot and cold E3 beakers also contained 1% v/v 1,2-propanediol. **(B)** The experimental protocol consisted of 6 min baseline recording, 7 min tricaine or gradual cooling application, and 50 min of recovery. **(C)** The heartrates of larvae exposed to a standard tricaine concentration (168 mg/L), gradual cooling (11°C) and control fish. Average heart rates are shown, the SEM is shown as shaded envelope. Bars above time points indicate significant differences vs. time-matched control larvae (Tukey's HSD test, for clarity exact *p*-values are not shown, all *p* < 0.05, *n* = 7–8).

A modified stage was used in order to deliver anesthetic to the larvae (Figure 2A, Supplementary Figure 1). The stage was made from aluminum, with two rails (M-SP-3, Newport, Irvine, CA, USA) onto which custom-made aluminum blocks were loaded, which functioned as heat-sinks. A circular Peltier element (TEC-15,2-6,0-51,0-71-51/9-RCH, Minkin Arctic TEC Technologies, Dortmund, Germany), with a hole in the middle was placed on top of the set-up. This hole allowed the eye movements and heartbeat frequency to be recorded from below. A modified glass-bottomed petri dish was fastened above the Peltier element (GW5040B-01, Plano, Wetzler, Germany). Hot glue was used to modify Petri dishes such that E3 medium could flow through only a small channel across the dish. Two tube connectors were glued to each side of the petri dish, and E3 medium was constantly added on one side of the dish and removed on the other side of the dish (P-801, Techlab, Braunschweig, Germany). The connectors removing E3 medium were placed at a shallower angle than those which added E3 to ensure a column of E3 medium remained at all times (~15° vs. ~30°). The E3 medium was provided by a peristaltic pump (ISM4408, Reglo Ismatic Digital, Cole-Palmer, Wertheim, Germany) with a flow rate of 15 ml/min. An overview of anesthesia application is shown in the schematic in Figure 2A. For experiments where tricaine was used, one tube supplied E3, another supplied E3 mixed with a known concentration of tricaine, and the other two were used for removal of E3 from the dish. As previously stated, for all cooling experiments the E3 used contained 1% v/v 1,2-propanediol (141545.1211, AppliChem, Darmstadt, Germany) to prevent freezing of the E3 medium at lower temperatures. For these experiments, one tube supplied E3 heated to 60°C, the other supplied cold E3 from a beaker containing ice made from E3 medium, again two tubes were used to remove E3. After passage through the tubes, which resulted in passive warming of the cold E3 and passive cooling of the hot E3, the solutions arrived and mixed in the dish. Temperature was tracked in real time using a Ni-Cr temperature probe and digital thermometer, and sent via a DAQ device in order to dynamically control the temperature (NI USB-6008, National Instruments, Austin, TX, USA).

The Peltier element was used during behavioral OKR and heartrate testing, but not during calcium imaging experiments. The same 5V DAQ device was also connected to three LED drivers (RCD-24-0.35/PL/B, Recom, Gmunden, Austria) which were connected in parallel and powered the analog dimming function and thus altered the current supplied to the Peltier based on the desired temperature. The power supplied to the Peltier was kept constant throughout the 7 min exposure period. The Peltier was only connected to a power supply during the cooling periods.

Removable aluminum blocks were used as heatsinks during the experiment. The blocks were kept in the freezer at −20°C and two blocks were added to the setup immediately before the beginning of the experiment. In the case of behavioral OKR and heartrate experiments, these blocks were exchanged every 20 min. This was not possible during calcium imaging experiments, and instead the blocks were exchanged during the pause between the first and second recording. The addition of the blocks lead to a reduction in temperature of the set-up prior to the start of the experiment and many of the fish were briefly exposed to temperatures of 16–20°C during the alignment of the fish. As this only occurred during the experiments where cooling was carried out (i.e., cooling experimental and cooling control groups), and since we did not observe any significant differences between baseline values for the four conditions (2 controls and 2 treatments), we assume it is unlikely that the early addition of the blocks influenced our findings.

**Figure 3:**
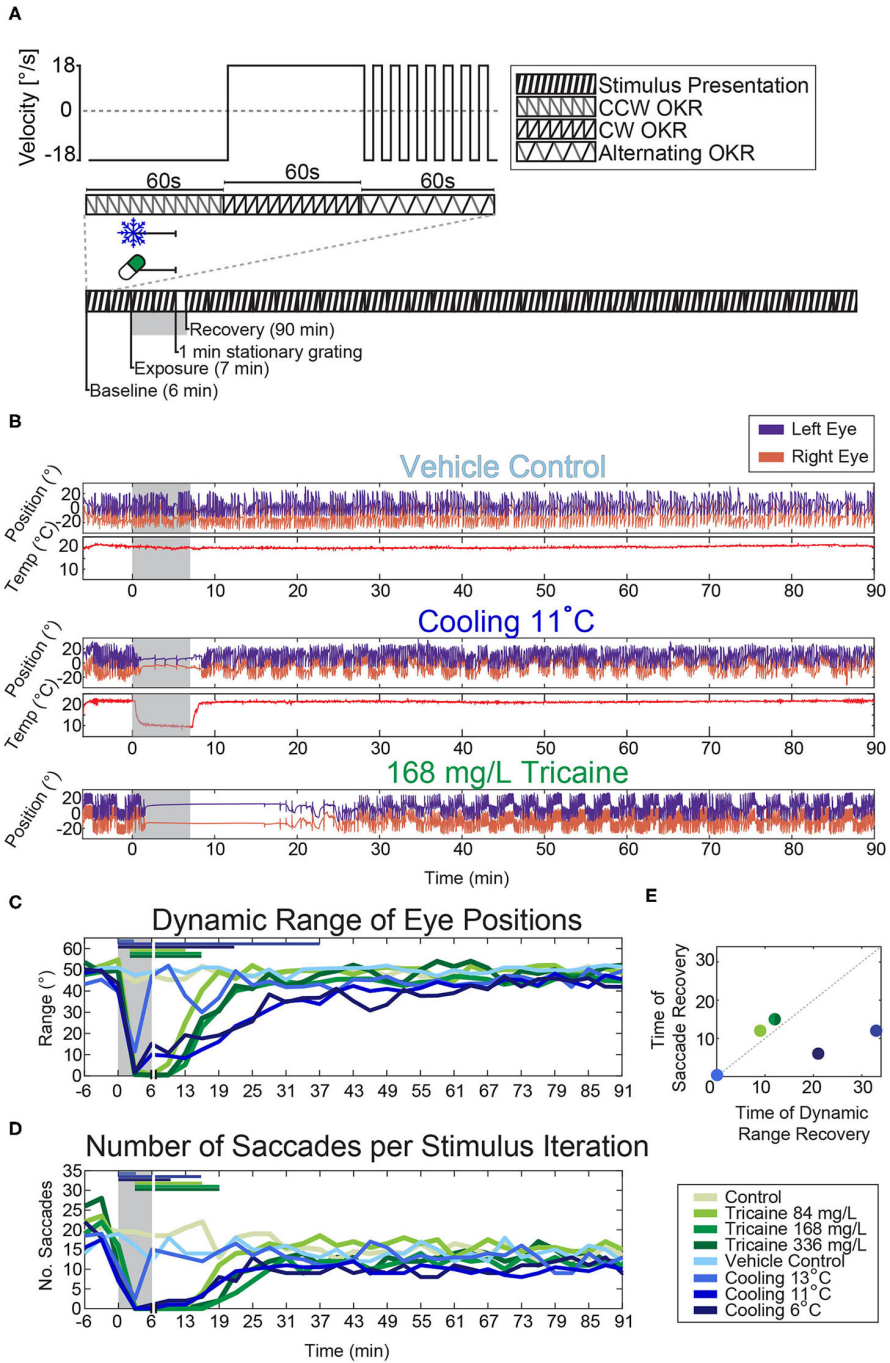
Effects of tricaine and gradual cooling on the optokinetic response. **(A)** Illustration of the visual stimulus (top) and the experimental protocol (bottom). A 3 min looping stimulus was presented to animals using an LED arena (Supplementary Figure 1). It consisted of a moving bar rotating at a constant velocity (18°/s) for 60 s counter-clockwise, followed by 60 s clockwise, and finally alternating between counter-clockwise and clockwise in 4 s intervals for a total of 60 s. **(B)** Sample traces of the eye movements evoked by the stimulus protocol during experiments carried out in the presence of the vehicle control (1% v/v 1,2-propanediol), during gradual cooling (11°C) and in the presence of tricaine at a concentration of 168 mg/L. **(C,D)** The dynamic range of the evoked eye movements and the number of saccades occurring during each 3 min stimulus period were analyzed and significance was determined via two-way repeated measures ANOVA with Tukey's HSD test. Horizontal colored bars indicate which time points were significantly different from respective controls, for clarity only one significance level is shown (*p* < 0.05). Vertical white lines indicate the 1 min stationary grating period shown in **(A)**, as no visual stimulus was present during this time. **(E)** Comparison of the recovery timepoints of saccade rate and dynamic range for the different treatments. *N* = 4–18.

The program ZebEyeTrack was used to control the peristaltic pump while also detecting and tracking the eyes, and displaying visual stimuli using an LED visual stimulus arena (26). The visual stimulus used during behavioral experiments consisted of a moving bar stimulus with a spatial stimulus frequency of 0.033 cycles per degree and a temporal frequency of 18°/s. The stimulus had three phases: 1 min counter-clockwise rotation, 1 min clockwise rotation, and 1 min of 4 s alternations between these two directions (see Figures 2B, 3A). The data was binned in 3 min increments and analyzed based on the number of saccades occurring, and the dynamic range of the eye movements observed. The dynamic range was calculated as the difference between the leftmost and rightmost extreme eye position. Significance was determined via 2-way repeated measures ANOVA with Tukey's honest significant difference (HSD) test, *n* = 4–18.

### Heartrate Measurement Alongside OKR Testing

Experiments were carried out as described above, though with a shorter format; recovery was recorded for 50 min only (see Figure 2B). During these experiments, the camera below the fish recorded a video of the heartbeat throughout the complete experiment. The frame rate of these recordings was 7–15 frames per second (fps). Videos were rotated in the image processing package Fiji (27), kymographs were generated for both the heart, and another area of the video in order to capture both the heart rate and background noise. The background noise signal was removed from the heartrate trace via an adaptive recursive least square filter. The heartrate signal was then resampled to a constant framerate of 10 Hz and filtered using a high pass filter with a cut-off frequency of 0.5 Hz (visual observation confirmed that there was no cessation of heartrate during any treatment). The Fourier synchro-squeezed transform of the trace was then calculated (Matlab: *fsst*); a simple Fourier transform was not performed due to the irregular sampling rate and the expected variability in heartrate during the recordings. The temporal frequency ridge was then extracted to determine the maximum energy frequency of the recording. Smearing was observed in the temporal frequency ridges immediately after treatments were applied. In order to remove this smearing, an inverse Fourier synchro-squeezed transform (Matlab: *ifsst*) was performed using the highest energy components of the signal (Kaiser window 256, β = 10), and the power spectrum of the resulting reconstructed signal was calculated (Matlab: *pspectrum*). The temporal frequency ridge of this reconstructed signal was taken as the heartrate (Matlab: *tfridge*). Controls were pooled; 2 unaltered-E3 controls were recorded, 4 controls with E3 containing 1% v/v 1,2-propanediol were recorded. Data was analyzed via 2-way repeated measures ANOVA with Tukey's honest significant difference (HSD) test, *n* = 7–8.

### Calcium Imaging Alongside Visual Stimulation

The two-photon imaging path and LED visual stimulation arena have been previously described in Brysch et al. (28). Calcium imaging was performed using a MOM microscope (Sutter Instruments, Novato, CA, USA), Coherent Vision-S Ti-Sa laser and a 20x/1.0 objective (Zeiss W Plan-Apochromat, Jena, Germany). Imaging was carried out at a frequency of 2 Hz, a magnification of 2x in the pretectum, and 1.3–1.5x in the hindbrain, and at a wavelength of 920 nm. Only one plane was imaged per fish per experiment. This was followed by sequential imaging of each brain structure in planes along the optical axis (z-axis); images were taken in intervals of 0.88 μm covering the brain volume 40 μm above and 40 μm below the target image plane (z-stack). *N* = 52 total fish were imaged.

**Figure 4:**
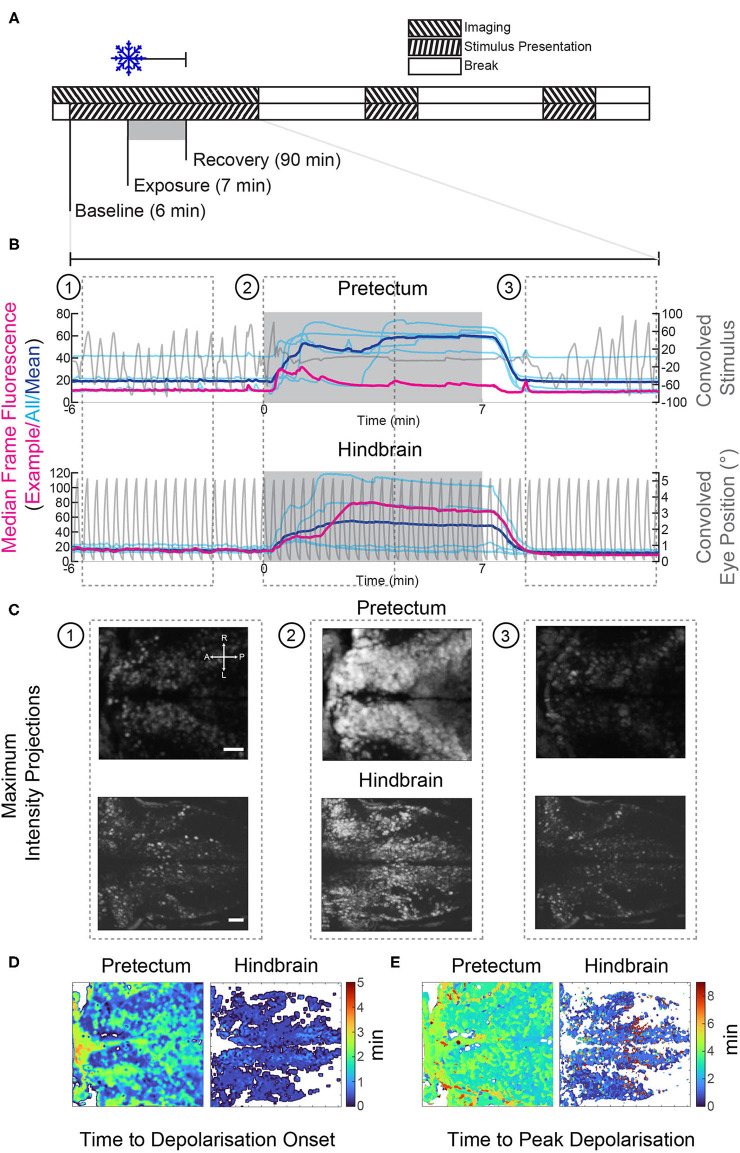
Gradual cooling induced a calcium surge in both the pretectum and hindbrain of exposed larvae. **(A)** Stimulus and imaging paradigm for calcium imaging experiments carried out in both the pretectum and hindbrain. **(B)** Median frame fluorescence for pretectal and hindbrain recordings; traces for individual recordings are shown in cyan and the mean values are shown in blue. The fluorescence trace for a single recording in the pretectum and hindbrain is shown in red, the convolved eye trace and convolved stimulus for the example recordings are also shown in gray. **(C)** Maximum intensity projections at different time points from the example recordings shown in red in **(B)**. 1: before cooling, 2: during cooling to 11°C, 3: recovery 1:30 to 5:40 min after cooling ceased. Scale bars: 30 μm. **(D)** The onset of the calcium surge was calculated in stimulus-correlated (regressor score >0.5) pixels. This onset was defined as the first time point at which the fluorescence was >5 standard deviations above the baseline. **(E)** The time point at which correlated pixels reached their highest pixel intensity. A, anterior; P, posterior; L, left; R, right.

**Figure 5:**
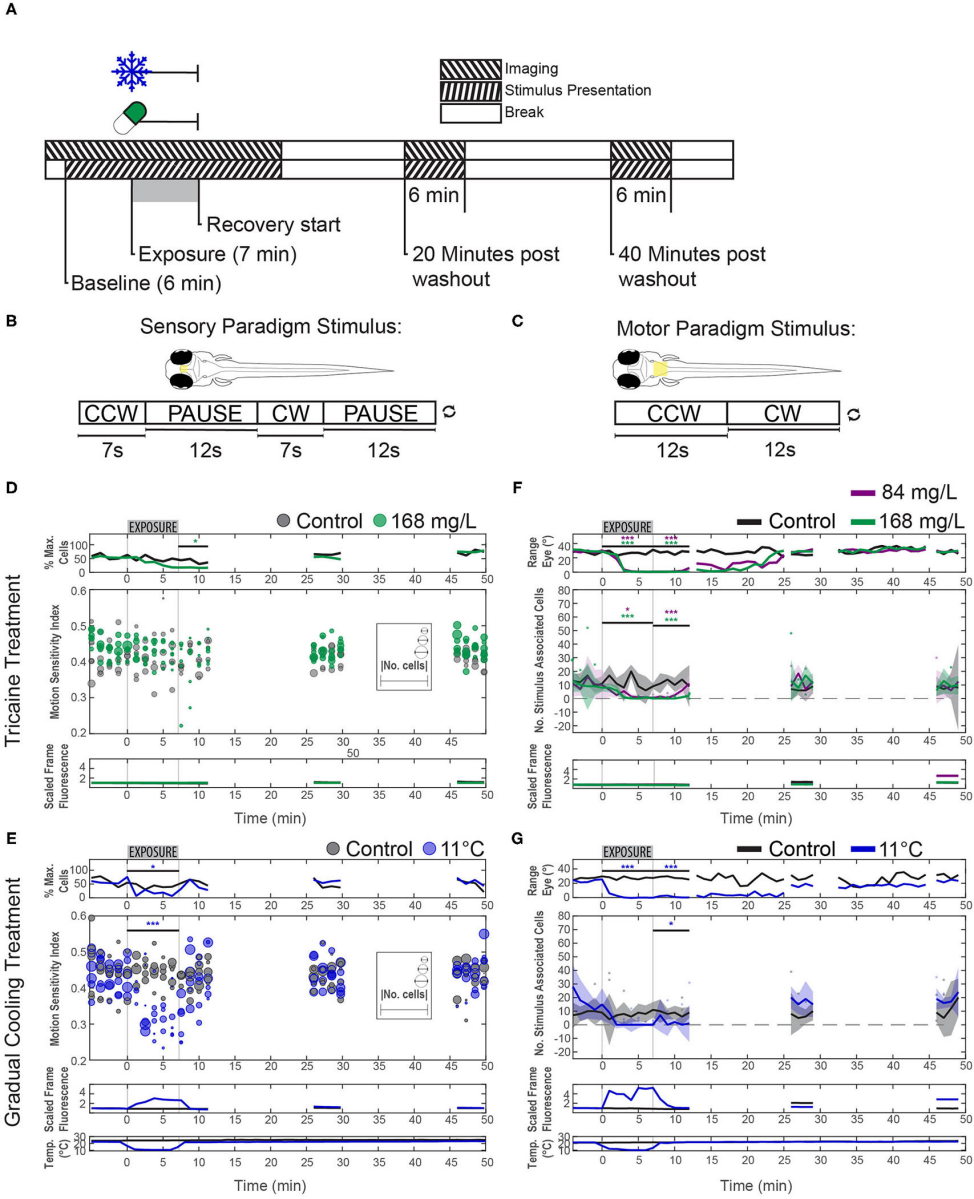
Effects of tricaine and gradual cooling on sensory and motor brain areas. **(A)** The duration of drug exposure was the same as in our previous experiments. Stimuli were presented alongside calcium imaging, and ceased between recordings, as shown in the schematic. Tricaine (168 mg/L and 84 mg/L) and gradual cooling treatments were applied for 7 min (Exposure). **(B)** During imaging of the pretectum, the stimulus consisted of alternating moving (7 s) and stationary (12 s) periods in order to determine the motion sensitivity of identified neurons. **(C)** For experiments in the hindbrain, the stimulus was constantly rotating (18°/s) and alternated between clockwise and counterclockwise motion every 12 s. **(D,E)** Motion-sensitivity of neurons detected in larvae exposed to tricaine and gradual cooling. Note that cooling treatment reduced the number of detected motion-sensitive cells and their motion-sensitivity, while the corresponding effects during tricaine treatment were less pronounced or absent. In the upper row (% Max. Cells), the number of detected neurons is expressed as a percentage of the maximum number of neurons detected via pixel-wise regressor correlation in any minute of the recording. The absolute number of identified cells in individual recordings is shown via the diameter of the data points. **(F**,**G)** The number of stimulus-associated neurons detected in the hindbrain of larvae exposed to either tricaine or gradual cooling was decreased. The dynamic range of eye positions is shown in the top row. Plots in **(D–G)** differ in style, because in **(D,E)** we assessed two parameters (motion sensitivity and number of cells, each represented by the circles) and only one parameter (number of cells) in **(F,G)**. Results were binned per phase into baseline, treated, recovery, recording 2 and recording 3 and analyzed via two-way repeated measures ANOVA with Tukey's HSD test, *n* = 4–7. *p < 0.05, ***p < 0.001.

For these experiments the optimal temperature, as determined from behavioral experiments to be 11°C, was tested against the standard tricaine concentration of 168 mg/L. Separate controls were used for comparison; a drug-free control group was tested vs. tricaine, and a vehicle (1% v/v 1,2-propanadiol) control group was compared with the cold treatment. For each experiment, an initial 2 min period of spontaneous activity was recorded, followed by a 6 min period of visual stimulation pre-anesthesia recording, this was then followed by a 7 min period of anesthesia alongside visual stimulus, and another 6 min period during which the anesthetic was removed alongside visual stimulus to show the initial recovery. After a 12 min break a subsequent 6 min recording was made, followed by a 13 min break, and another 6 min recording. A moving-bar visual stimulus was shown during these recordings, but remained stationary during the breaks (see Figures 4A, 5A).

Visual stimulation experiments were carried out alongside calcium imaging, using two distinct stimulus protocol paradigms, one aimed to identify and characterize sensory neurons and the other to do this for motoneurons (see Figures 5B,C).

During analysis, we observed that in the neural recordings, where gradual cooling treatment was used, the plane of the recordings underwent a drift in the z-plane, likely owing to the cooling and heating of the aluminum stage. The linear thermal expansion coefficient of aluminum is ~23^*^10^−6^/K, therefore for a stage of this height (15 cm) a 1°C change in temperature would result in a shift of 3.45 μm. Additionally, there was a dramatic alteration in the appearance of the frames, which may have been due to cellular swelling and could alter the pixel identities of neurons in the recording. As these effects were exclusively observed during cooling treatment, this could have caused us to misattribute changes in firing patterns which were due to cells drifting or expanding as being caused by anesthetics. To minimize this possibility, rather than analyze recordings *in toto* we instead broke them up into smaller fragments of 60 or 75 s (for motor hindbrain and sensory pretectum recordings, respectively). Regions of interest (ROIs) were then automatically generated in each fragment in order to identify stimulus encoding neurons in both sensory and motor brain areas. ROIs consisted of 15–25 spatially contiguous pixels whose z-scores were highly correlated with the chosen regressor.

### Pretectal Imaging

Imaging was carried out in the pretectum, directly beneath the dorsal boundary to the optic tectum, in order to look for deficits in sensory signals during anesthesia. The visual stimulus consisted of a 7 s exposure to a moving bar stimulus, interspersed with a 12 s pause (see Figure 5B; sensory paradigm). After each pause, stimulus direction (clockwise, counter-clockwise) reversed. This stimulus was repeated throughout the recordings, but was not shown between recordings. All video fragments underwent analysis with a previously published method in order to detect ROIs based on pixel-wise correlation with a stimulus encoding regressor (28, 29). The motion sensitivity index of all identified ROIs was calculated based on the activity of the neurons during moving vs. stationary stimulus periods for two full stimulus presentations; if the index fell below 0.1 in more than two of the four iterations the ROIs were excluded from further analyses. This step was implemented to control for false positive motion responses caused by a thermally induced calcium surge.

The first four of the final 5 s of the stimulus presentation were used (t_end−5_: t_end−1_).

The calcium signal DFF at time point t was calculated as the difference between the calcium indicator fluorescence and the baseline calcium indicator fluorescence (Fb; defined as the mean of the lowest 25 values in the ROI trace) divided by Fb:


(1) $$\begin{array}{r} DFF\left(t\right)=\;\frac{F\left(t\right)-Fb}{Fb} \end{array}$$

The motion sensitivity index of the j^th^ ROI was calculated as follows:


(2) $$\begin{array}{c} MSI_{j}=\;\frac{\sum_{t}DFF_{j}on(t)-DFF_{j}off(t)}{\sum_{t}DFF_{j}on(t)+DFF_{j}off(t)} \end{array}$$

where DFF^on^ (t) and DFF^off^ (t) correspond to the calcium signal during static and moving stimulus presentation, respectively.

Data was analyzed via two-way ANOVA with Tukey's honest significant difference (HSD) test, *n* = 5–6.

A control experiment was carried out during which larvae were recorded for 6 min prior to the addition of tricaine followed by exposure to 168 mg/L tricaine for 15 min. The visual stimulus was shown throughout. A 1-way ANOVA was carried out on detected ROIs across 75 s time-bins on the resulting motion sensitivity values, the results of which were not significant, *n* = 4. A 1-way ANOVA on the number of ROIs detected across time-bins was highly significant (*p* < 0.001). This control experiment showed that a longer treatment period (15 min) had similar effects as the shorter treatment period used in all other recordings. In both type of experiments, tricaine reduced the number of detected ROIs, but not the motion sensitivity of these remaining ROIs. Therefore, the finding of remaining motion sensitivity does not appear to be related to the shortness of our treatment period.

### Hindbrain Imaging

Imaging was carried out in the zebrafish hindbrain in order to detect deficits in motor signals during anesthesia. Recordings were carried out in the plane corresponding to the location of the Mauthner cell somata and extending rostrally to the cerebellum and caudally to the spinal cord, the imaging region extended dorsally for 20 μm. The visual stimulus consisted of a moving bar stimulus, which alternated between clockwise and counterclockwise every 12 s, but was not shown between recordings (see Figure 5C). The analysis of motor encoding activity was complicated by the loss of eye movements during anesthesia. In order to determine whether residual motor encoding signals were present during periods where no behavior was seen, our approach focused on neurons whose firing was modulated by the stimulus. Due to the sinusoidal nature of the stimulus, a fast Fourier transformation was carried out on the detrended DFF traces, and the number of ROIs which had a peak at the stimulus frequency was counted. Changes in the number of identified cells were analyzed via two-way ANOVA with Tukey's honest significant difference (HSD) test, *n* = 4–7. Wilcoxon rank-sum tests were used to determine changes in the median dynamic ranges as data were found to be non-parametric.

### Cross-Correlation Structures

ROIs detected in each time bin were ordered based on their correlation with the clockwise-stimulus regressor. The resulting matrices were then averaged within each of five treatment stages (pre, during, post and two recovery stages) resulting in five correlation maps per larva. A weighted average was then generated across larvae based on the number of ROIs identified.

### Generation of Calcium Surge Heat Maps

Heat maps shown in Figures 4D,E were generated for whole recordings of gradual cooling experiments. 8-bit rigid body registered AVI recordings were used. The videos were first filtered using a two-dimensional Gaussian filter (σ = 2) (Matlab: *imgaussfit*). Individual pixels were then smoothed using a Butterworth filter (1st order, cut-off frequency 0.2 Hz; Matlab: *butter*), as described by Niemeyer et al. (30). Individual pixels were correlated with the median fluorescence trace in order to generate a pixel correlation mask, only pixels with a correlation >0.5 were included in this mask. The onset of the calcium surge was determined as the time point at which the pixel value exceeded five standard deviations above the baseline. A Wilcoxon rank-sum test was carried out in order to determine whether the surge occurred significantly earlier in the hindbrain vs. the pretectum. The onset values for all pixels contained within the masks for all recordings in either the hindbrain or pretectum were collated and the resulting populations were statistically tested against one another. The maximum calcium surge was determined as the time point at which the pixel value was highest. In order to exclude outliers, this value was only included if the threshold was surpassed a second time within 3 s.

## Results

### Behavioral Effects in Free Swimming Larvae

All larvae exposed to tricaine (Figure 1A) rapidly lost their tactile response (Figure 1B; median durations of 43 s for a concentration of 84 mg/L, 25 s for 168 mg/L, and 15 s, for 336 mg/L). This was followed quickly by the loss of righting reflex (Figure 1C; median durations of 46 s for 84 mg/L, 28 s for 168 mg/L, and 15 s for 336 mg/L) indicating that the fish had reached surgical level anesthesia [stage 3 as defined by Sneddon (31)]. The time to recovery, as assessed by response to tactile stimulation, was 85 s for 84 mg/L, 147 s for 168 mg/L, and 212 s for 336 mg/L. Thus, anesthesia onset was much faster than recovery and both onset and recovery in free swimming larvae were strongly dose dependent as assessed via 1-way ANOVA (tactile response *p* < 0.001; equilibrium *p* < 0.05; recovery *p* < 0.001).

### Effects of Anesthetic Agents on the Optokinetic Response

In order to analyze the effects of anesthetics on behavior we assessed the optokinetic response. The optokinetic response is a reflexive behavior and therefore we assume that it is harder to suppress by anesthetic agents than, for example, appetitive behaviors like prey capture responses. Thus, it should provide an accurate readout for the general effects of anesthesia on behavior and brain responsivity. The anesthetic agents were applied via a peristaltic pump. Three tricaine concentrations and three temperatures were tested. In the case of cooling, hot and cold E3 medium, containing 1% v/v 1,2-propanediol, were mixed within the Petri dish to achieve one of the three defined temperatures; a thermometer fixed in the dish allowed for dynamic control of the ratio of hot to cold water in the dish (see Figure 2A). In the case of tricaine application, E3 medium containing tricaine at one of the test concentrations was pumped into the Petri dish and later washed out and thereby replaced by medium not containing tricaine (Figure 2A).

During each of the experiments the agarose surrounding the eyes was removed and animals were stimulated with a moving grating presented on a surrounding display to elicit an optokinetic response (Figure 3A). The effects of each method of anesthesia on the dynamic range of the eye position (Figure 3C) and the number of saccades (Figure 3D) were assessed. A two-way repeated-measures ANOVA, with Tukey's HSD test was carried out on the resulting data. Both the effects on the dynamic range and saccade numbers were significantly time-dependent (*p* < 0.001 for both tests), and treatment-dependent (*p* < 0.001 for both tests). Larvae exposed to tricaine recovered their dynamic range of eye movements before saccades returned, the opposite was true for gradually cooled larvae (Figure 3E).

Tukey's HSD tests found that the dynamic range of eye movements was significantly decreased from about 30° to <10° after 3 min of exposure to either anesthetic (Figure 3C, Supplementary Figure 2). The onset was faster for the cooling treatments. Considering our earlier results that tricaine is effective within 1 min in free swimming larvae (cf. Figure 1B), this difference in effect onset timing is likely due to the time taken for the tricaine to reach an effective concentration in the agarose-embedded larvae. The full recovery of dynamic range occurred faster in tricaine-treated animals (ca. 10 min) than in animals cooled to 11 or 6°C (ca. 15 to 30 min), while larvae cooled to 13°C recovered almost instantly.

In cooled larvae, the saccade rate was reduced to almost zero saccades per minute after 3 min, and appeared to recover within 5 min. The loss of saccades was longer-lasting in larvae treated with the standard tricaine concentration (168 mg/L) than in cooled larvae and remained significant for up to 14 min following removal of tricaine. In cold-exposed larvae, the recovery success was more variable across larvae, whereas tricaine-treated larvae showed less inter-individual variability (data not shown).

### Zebrafish Heartrate

The effects of anesthetics on the heart rate were analyzed by applying the two anesthetic agents via a peristaltic pump (Figures 2A,B). We chose a temperature of 11°C because it was shown to be effective in the optokinetic response experiments just described. The standard concentration (168 mg/L) of tricaine was used.

These experiments showed that cold and tricaine treatments have temporally distinct effects on zebrafish heart rate (see Figure 2C, Supplementary Figure 3). Both experimental protocol time and treatment type had significant effects on heartrate as assessed via a repeated-measures ANOVA (*p* < 0.001 for both conditions). Tricaine did not appear to alter the heartrate in the treatment period, which agrees with previous reports (32). However, a heartrate increase was seen during recovery commencing at the time point corresponding to the time at which eye movements (see below) had fully recovered to control levels (~22 min, see Figure 2C), though this was not significant throughout most of the recovery period. Gradual cooling by contrast had profound effects on heartrate, causing a 60 % drop in heartrate during treatment, followed by a strong and sustained increase (~15%) during recovery.

### Neuronal Signaling in Sensory and Motor Brain Areas

We measured neuronal activity via calcium imaging and used a similar experimental protocol as before, but with a modified visual stimulus protocol (Figures 5B,C) to best capture alterations in the responses of neurons in the pretectum and hindbrain.

In all cooling experiments, regardless of brain area, the calcium level of the neurons started to increase drastically within ca. 30 s of cold treatment initiation and then remained elevated (Figures 4B–E). Calcium levels dropped once the temperature returned to baseline levels (Figure 4B), i.e., within 2 min after the end of the cold treatment period. Fluorescence changes were directly linked to our recorded temperature changes, but slightly delayed (Figures 5E,G; bottom). The timing of calcium surge onset was somewhat variable across neurons (Figures 4B,D,E), and occurred significantly earlier in hindbrain (mean onset 32 s, standard deviation 28 s) than in the pretectum (mean onset 43 s, standard deviation 35 s) (Wilcoxon rank-sum test, *p* < 0.001). Despite these drastic fluorescence changes, we were able to detect and quantify stimulus-associated neural activity which rode on top of the calcium surge (Supplementary Figure 4, also discussed below).

**Figure 6:**
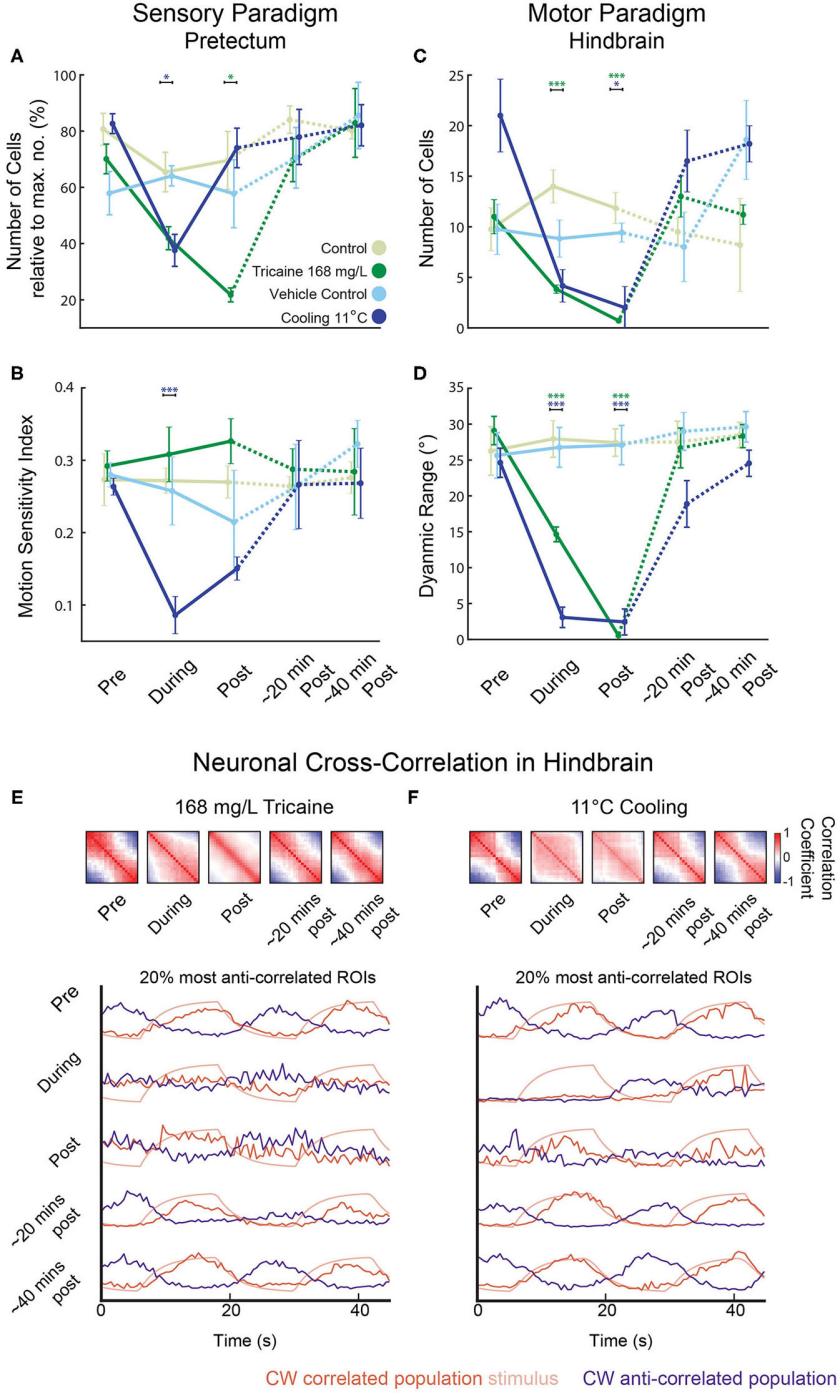
Reduced sensorimotor activity in pretectum and hindbrain after application of tricaine and gradual cooling anesthesia. **(A–D)** Summary of the results from Figure 5 highlighting the main treatment effects on the number of identified cells in the pretectum **(A)**, the motion-sensitivity index of remaining pretectal neurons **(B)**, the number of identified cells in the hindbrain **(C)**, and the dynamic range of eye movements observed **(D)**. In **(A)**, the number of remaining motion-sensitive neurons relative to the maximal number of motion-sensitive neurons detected in the recording is quantified. Results were binned per phase into baseline, treated, recovery, recording 2 and recording 3 and analyzed via two-way repeated measures ANOVA with Tukey's HSD test, *n* = 4–7. *p < 0.05, ***p < 0.001. **(E,F)** Top: Pair-wise neuronal cross-correlation matrices for the hindbrain of larvae treated with 168 mg/L tricaine or cooled to 11°C. y and x axes of each matrix correspond to the neuronal ROIs sorted according to their individual correlation to the clockwise stimulus. Cross-correlation matrices were calculated for the five different time periods shown and in each displayed matrix, the matrices of all individual recordings and larvae were averaged (a weighted average that took into account the number of ROIs per recording). Bottom: The 20% most correlated (positively or negatively) ROIs are shown. Stimulus traces show expected activity of a CW stimulus-correlated ROI (i.e., the kinetics-adjusted stimulus regressor see Methods).

Calcium signals were analyzed in fragments of 60 s each (see Methods) to circumvent artifacts resulting from image drift. The image drift was likely caused by a combination of temperature-dependent changes of the aluminum stage (linear thermal expansion coefficient: 0.002 % per Kelvin) and also morphological changes of the larva induced by the cooling. Tricaine and gradual cooling differed in their effects on sensory tuning (see Figures 5D,F, Methods), which we quantified using a motion-sensitivity index and the number of remaining motion-sensitive neurons (see Methods). Next to detailed plots showing results for all individual recordings (Figures 5D,F), the main findings are illustrated in the summary plots in Figures 6A,B. Treatment had a significant effect on the motion sensitivity index over time (2-way repeated measures ANOVA, *p* < 0.05) and time alone (*p* < 0.001). In gradual cooling experiments, the number of detected neurons with stimulus-associated activity dropped significantly during treatment (Tukey's HSD test, *p* < 0.05), but recovered within 5 min after return to baseline temperature (Tukey's HSD test, *p* = 0.6). During tricaine experiments, the number of detected stimulus-associated ROIs was not significantly altered during treatment (Tukey's HSD test, *p* = 0.09), but became reduced during the first 5 min of recovery (Tukey's HSD test, *p* < 0.05). The number of detected cells was significantly correlated with treatment (*p* < 0.001) and time (*p* < 0.001) in 2-way ANOVA.

In contrast to the change in the number of detectable stimulus-associated neurons, detected neurons still showed normal levels of motion sensitivity during tricaine treatment (no statistical difference observed, *p* = 0.78, Figure 5D), and direction sensitivity was also unaltered (data not shown). For the cooling treatment, a reduction in measured motion sensitivity was observed (Tukey's HSD test, *p* < 0.001). This apparent loss of motion sensitivity in the cooling condition was likely—at least in part—caused by the effects of the calcium surge in our analysis. An independent analysis, which relied on image analysis to detect ROIs (33), was performed to exclude potential analysis bias resulting from the calcium surge, also detected stimulus-associated pretectal calcium responses, which appeared to be lost during cooling but to recover afterwards (Supplementary Figure 5).

To determine whether the only moderate effects of tricaine treatment on sensory brain activity were due to the short exposure period, four larvae were exposed to 168 mg/L tricaine for 15 min and a 1-way ANOVA was carried out to determine whether the stimulus-associated activity was altered. This test did not find an according significant effect. While the number of detected neurons also decreased over time in these extended recordings, stimulus-associated neurons were found in all but the final minute of one of these four recordings. Thus, tricaine-anesthetized zebrafish show residual sensory brain activity with relatively normal tuning although optokinetic behavioral responses have ceased.

Next, we characterized premotor and motor hindbrain responses in the caudal hindbrain. This brain region contains neuronal populations which drive the lateral and medial rectus extraocular eye muscles, including abducens motoneurons, abducens internuclear neurons and further premotor neurons. We refer to these neurons as premotor from hereon, although motoneurons were likely included in our recordings. Both tricaine and gradual cooling had a pronounced effect on the number of stimulus-modulated neurons detected in the hindbrain (see Figures 5F,G, 6C,D); a 2-way ANOVA identified a significant influence of both time (*p* < 0.001) and treatment condition (*p* < 0.001). This decrease in the number of stimulus-modulated neurons was significant for both concentrations of tricaine during the treatment phase (168 mg/L *p* < 0.001; 84 mg/L *p* < 0.05) but not significant during cooling treatment (11°C). In the first minutes of recovery the number of stimulus-modulated neurons was decreased both for tricaine groups (168 mg/L: *p* < 0.001; 84 mg/L: *p* < 0.001) and for the gradual cooling group (11°C: *p* < 0.05).

There was also a significant effect of treatment on the dynamic range of eye positions (see Figure 6D); once again, a 2-way ANOVA identified a significant influence of both time (*p* < 0.001) and treatment condition (*p* < 0.001). This was significant for both tricaine (168 mg/L and 84 mg/L) and cooling treatment (11°C) during (*p* < 0.001) and in the recovery immediately after (*p* < 0.001). These results are in line with the previous behavioral findings (see Figure 3C).

### Loss of Coordinated Oculomotor Hindbrain Activity in Treated Larvae

In untreated larvae, the hindbrain generated alternating leftward and rightward oculomotor activity patterns associated with optokinetic responses to our alternating directions of stimulus motion. To characterize this premotor activity in treated animals (who had lost their ability to move the eyes), we quantified the pairwise cross-correlation between all neuronal ROIs in the hindbrain. Cross-correlations during each of the treatments differed from those immediately after treatment. Figure 6E shows cross-correlation matrices where ROIs were ordered based on their individual correlation to a clockwise-stimulus regressor. During both the spontaneous activity prior to the baseline period (not shown) and the baseline period before anesthesia application, this cross-correlation resulted in a structure with two observable anti-correlated populations (Figures 6E,F; red and blue). These anti-correlated populations mainly correspond to eye position drive in the left and right hemisphere of the hindbrain (especially the nucleus abducens), which code for leftward and rightward eye positions, respectively (28). During anesthesia, the neural activity lost its anti-correlational components. In the case of tricaine, there was first an increase in the number of pairs with positive correlations during treatment but immediately after treatment, excluding the diagonal of auto-correlation scores of 1, the positive cross-correlation matrix values were reduced. In comparison, in the case of cooling, negative activity correlations were lost as well for both treatment and early recovery, and most neuron pairs showed weak positive correlations during both treatment and early recovery. The deficit in anti-correlated activity, combined with the near-complete loss of stimulus-associated neurons (Figures 5F,G), suggests that during each treatment, the ability of the hindbrain premotor structures to encode bidirectional eye movements is strongly impaired, as the network activity becomes decorrelated.

## Discussion

**Figure 7:**
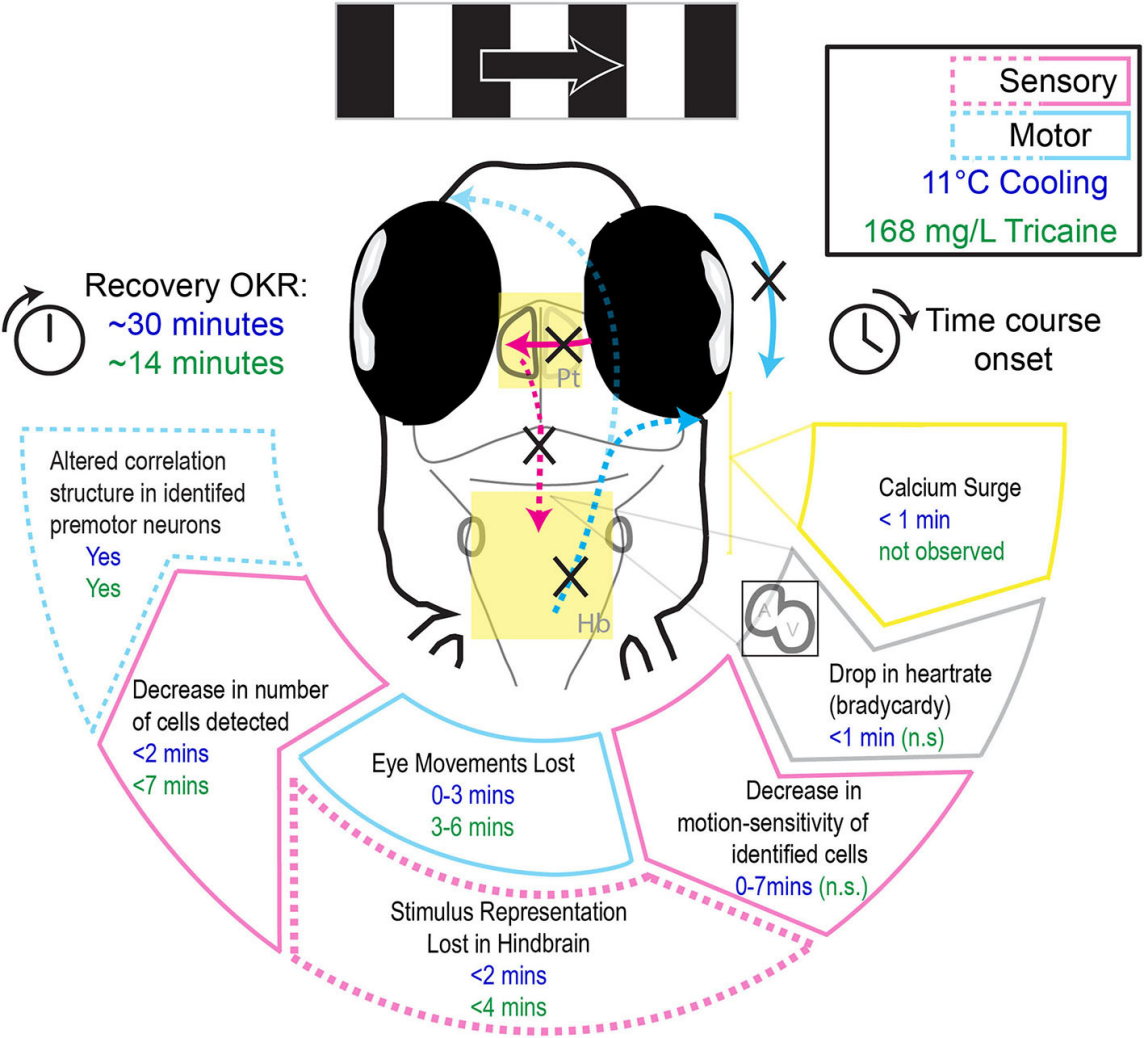
Schematic illustrating the differential effects of 168 mg/L tricaine and 11°C cooling on physiological parameters and the optokinetic response, as well as their respective time courses.

In this study we characterized the effects of two anesthetic agents, tricaine and gradual cooling, on swimming behavior, heartrate, eye movements, and sensorimotor circuits underlying the optokinetic response. We find that while tricaine has a slower onset, both tricaine and cooling are effective methods of immobilization and both suppress sensory and motor neural activity. But they differ in effects relevant to animal well-being (Figure 7). In contrast to tricaine, gradual cooling strongly reduces the heartrate, induces a calcium surge in the brain, permits residual eye movements, and has a comparatively longer recovery period. These results together suggest that tricaine should be considered the preferred anesthetic agent out of these two methods for zebrafish larvae.

### Tricaine and Gradual Cooling Have Comparable Effect Onset Times

In freely swimming larvae, a standard tricaine concentration induced loss of tactile response within ca. 40 s (Figures 1B–D). For agarose-embedded larvae, eye movements were lost within ca. 1 min in cooled larvae (Figure 5G) and ca. 3 min in tricaine-treated larvae. These results together suggest that in freely swimming larvae, the anesthetic effects of both tricaine and cooling manifest within tens of seconds and that the slower onset time for our tricaine in embedded animals was due to the (relatively) slow diffusion of tricaine through the agarose. Previous studies in adult fish have shown the onset of tricaine to be faster than that of gradual cooling (18).

Freely swimming fish regained their ability to respond to a startle-inducing stimulus within 147 s after tricaine (168 mg/L tricaine) wash-out. This recovery was much faster than the ~11–14 min' recovery seen for optokinetic responses in embedded fish treated with tricaine. The large difference in recovery times suggests that the optokinetic response is more vulnerable to anesthetic agents than the startle response, since the time difference cannot be explained by slower diffusion or removal of tricaine in the embedded preparation.

### Tricaine Suppresses the Optokinetic Response More Reliably Than Gradual Cooling

During behavioral experiments, we found that both tricaine and gradual cooling effectively suppressed the optokinetic response. However, while both treatments appeared to have a smooth and rapid onset, the anesthesia in the cooled larvae was much more variable as evidenced by the number of saccades occurring in the second half of the cold exposure. When observing the eye traces of the cooled larvae, approximately a third show convergent and divergent slow velocity eye movements and saccades during the treatment (see Figure 3B), which cannot be explained by the stimulus. While these saccades may be true spontaneous saccades, such a preponderance of spontaneous vergence activity is atypical. It is also possible that they were due to surges in neural activity, and are better described as seizure-type behavior. The presence of these spontaneous movements during anesthesia is problematic as they could occur during surgeries or other procedures, and potentially lead to injury.

### Cooling to 11°C Is Sufficient to Suppress Reflexive Behavior

The optokinetic response recovered most rapidly from exposure to 13°C, however, incidence of saccades and dynamic range of eye movements were not completely suppressed when compared to treatment with 11 and 6°C temperature (see Figure 3C). As we found that 11°C was already effective in suppressing both parameters of the optokinetic response to a similar degree to that of tricaine, we chose to use this temperature in both the heartrate and calcium imaging experiments which followed. Additionally, Collymore et al. (18) observed that an adult fish cooled to 8°C died, while 10°C was an effective temperature. Thus, a temperature of 10–11°C is likely safer for the larvae (though no larvae died at either temperature). While we found that fish exposed to 6°C apparently recovered faster than those exposed to 11°C, a larger sample size for the group tested at 6°C would be needed to confirm this trend.

### Gradual Cooling, but Not Tricaine, Decreases Heartrate

The effects of tricaine and cooling on heartrate differed during treatment. Only cooling resulted in a decrease, but both treatments resulted in a post-treatment increase in heartrate during recovery. Contrary to our results, other authors have reported heartrate decrease due to tricaine, which can potentially be explained by differences in study design. Tricaine exposure had previously been shown to maximally decrease heartrate after 10 min (34), which is longer than our total exposure time. Craig et al. (32) only saw significant effects at a concentration of 250 mg/L, much higher than what was tested here (168 mg/L). The decrease in heartrate observed for cooling is likely directly caused by a change in membrane permeability and conduction of the pacemaker cells (35). In line with this, Gierten et al. (36) have shown that temperature is positively correlated with heartrate in zebrafish, although they did not test at low temperatures. Though the sympatho-vagal balance of larval zebrafish has not yet reached its adult state (37), decreases in heartrate have been shown to be sensitive to muscarinic antagonists, consistent with the hypothesis that they are mediated by the parasympathetic nervous system, while increases can be blocked via beta-adrenergic antagonists, consistent with a potential sympathetic drive (38). Therefore, the fact that both treatments induced a tachycardic state following anesthetic removal and corresponding to the time point of recovery of eye movements may suggest that treatments were aversive or stressful for larvae.

### Tricaine Treatment Decreases the Number of Stimulus-Associated Neurons in the Pretectum, but Does Not Alter the Tuning of Those Detected

We found that tricaine significantly reduced but did not fully abolish the number of motion-sensitive cells detected in the pretectum, while the remaining tuned cells showed a similar level of motion tuning as cells detected in the baseline period (Figures 5D, 6B). These neurons were detected despite the lack of detectable eye movements seen in previous recordings (Figure 3B). Thus, during tricaine anesthesia, behavioral responses cease even though visual stimuli are still processed in the pretectum. This result is similar to previous findings in *Xenopus* (13) as well as those of Machnik et al. (39), who found that visual responses were decreased, though not entirely absent in Mauthner neurons in adult goldfish exposed to 100 mg/L tricaine.

### Gradual Cooling Induces a Wide-Spread Calcium Wave in Both the Pretectum and Hindbrain

In the case of gradual cooling, the number of identified stimulus-associated neurons was decreased in the pretectum, and in contrast to tricaine, an apparent decrease in the fidelity of remaining encoding pretectal neurons was seen (see Figures 5E, 6B). The interpretation of our fluorescence measurements during cooling is potentially strongly affected by the observed calcium surge, which might mask remaining motion sensitivity, or falsely imply motion sensitivity, due to the precise time point of the calcium surge relative to the motion stimulus. We attempted to control for this by requiring all motion-selective neurons to exhibit motion selectivity indices above a threshold of 0.1 in at least two out of four stimulus iterations per time-bin, thus removing neurons which may have had a calcium surge coinciding with motion during just one iteration.

Though the effects of cold on neuronal signaling have been described in other animals (40), to the best of our knowledge, this calcium surge has not been reported for zebrafish elsewhere. However, a spreading depolarization has recently been shown to occur in larval zebrafish exposed to noxious heat (41), and also following extended (>25 min) mechanical suppression of heartbeat (21). Spreading depolarization refers to a slow spreading wave of depolarization which travels through the brain, is similar to a seizure, but occurs at a much slower timescale (2–6 mm/min). This phenomenon is thought to underlie migraines and traumatic brain injuries (42), and may correspond to what we are observing here.

Calcium waves in the hindbrain appeared to both occur earlier and reach their peak levels earlier (Figures 4B–E) than calcium waves in the pretectum. However, due to pixel saturation in some recordings, we cannot unequivocally confirm this. While it is possible that the timing difference represents physiological differences between hindbrain and pretectum, it could also be a consequence of the embedding procedure: during hindbrain recordings agarose was removed from around the eyes, thus exposing the brain directly to the cooled medium.

The calcium wave may be caused by energy insufficiency (42, 43). Metabolic processes are known to slow down with decreased temperature, referred to as universal temperature dependence (UTD) (44). Cellular ionic homeostasis relies on a balance between passive and active fluxes. In fish, the temperature coefficient (Q_10_) of these processes are known to differ, meaning that at colder temperatures either passive flux must be downregulated, or active flux must be rapidly upregulated in order to maintain homeostasis (45). If the rate of active, ATP-dependent, processes falls below the rate necessary to maintain homeostasis, the balance shifts and, without a corresponding alteration in passive flux, this can lead to a depolarizing shift in resting membrane potential intracellular sodium accumulation. The Na^+^/K^+^-ATPase, which is responsible for sodium extrusion, is the most energy demanding neural process (46), and if neurons do not have sufficient energy, they can no longer maintain osmotic balance. This increased intracellular sodium concentration will then result in cell swelling as water enters the cell via the osmolality gradient (47, 48). It also reduces the membrane potential, and causes the reversal of the Na^+^/Ca^2+^ exchanger resulting in Ca^2+^ entry to the cell and eventual depolarization (49, 50). The calcium ions continue to accumulate as the activity of the Ca^2+^ ATPase pump is also decreased, as is ATP-dependent reuptake by the endoplasmic reticulum. Failure of the Na^+^/K^+^-ATPase pump is thought to be the mechanism behind spreading depolarization, a phenomenon reported to occur during noxious heating of larval zebrafish (41). Similarly, it appears likely that the observed calcium wave in our experiments was caused by insufficient active efflux from neurons during cooling (50). Both decreases in passive flux and increases in active flux have been independently reported in long-term cold adapted fish (45) which may explain why zebrafish can inhabit colder water bodies [down to 6°C according to (51)], however, such adaptations likely occur over much longer time courses than those induced in this study.

ATP depletion has been widely considered to underlie the damaging effects of hypothermia, and the possibility that this is what occurs during cooling in our experiments is supported by three observations. First, the transgenic zebrafish *Tg (smyd1:m3ck)* exhibits a 2.16-fold higher ATP level and indeed maintains swimming behavior at temperatures of 13 degrees (52) vs. wildtype fish who cannot swim anymore, suggesting that energy availability may allow rapid changes in temperature to be tolerated. Second, following cold acclimatization, RNA transcripts involved in energy metabolism are upregulated, in particular those related to glycolysis (52, 53). Third, hypoxia treatment increases cold tolerance in zebrafish (54)—likely due to the resultant increased capability of zebrafish to undergo anaerobic respiration (55). These findings suggest that increased ATP availability and anaerobic respiration capabilities underlie cold acclimatization and tolerance. Indeed, lactate is found in the brains of cold-exposed zebrafish larvae (56), the end product of anaerobic respiration. Though larval zebrafish receive sufficient oxygen via diffusion to survive up to 6 dpf, active blood circulation increases oxygen uptake (57), and mutant larvae with bradycardia show signs of hypoxia and developmental retardation (58). Thus, the decrease in heartrate induced via cooling may be another factor in the shift of larval metabolism toward anaerobic respiration.

It is unclear whether the mechanism underlying the calcium surge is independent of the anesthetic effects, as it could potentially also underlie it. Should this not be the case, it still remains unclear whether this calcium wave could have been avoided by slowing the rate of cooling, or whether this would instead simply have delayed it. While it is possible that it may not have occurred, we believe that the cooling rate which was used here is in line with that of Collymore et al. (18), and can realistically be implemented in routine laboratory practice. Cooling rates used in the aquaculture industry are much slower [for example, 4°/h (59)], and thus incompatible with the workflows of most research laboratories carrying out experiments.

### Tricaine and Cooling Decrease the Number of Stimulus-Modulated Neurons in the Hindbrain

Both tricaine and cooling led to a decrease in the number of stimulus-modulated neurons found in the hindbrain (see Figures 5F,G, 6C). In our investigation of the pairwise cross-correlations of the activity of hindbrain neurons (Figures 6E,F), we observed a strong reduction of anti-correlations for both anesthesia treatments. These results suggest a loss of functional connectivity between the pretectum and hindbrain, as the remaining stimulus-tuned cells in the pretectum were no longer able to effect high levels of left-right alternating oculomotor activity in the hindbrain. Loss of functional connectivity is a defining characteristic of anesthesia (60–62), and a suggested mechanism behind loss of consciousness (63). Furthermore, both treatments appeared to increase the abundance of positive correlations, suggestive of a decrease in entropy, a reported characteristic of anesthesia. The use of alpha-bungarotoxin, a paralytic, has been shown to spare the anti-correlated-activity balance detected downstream in the spinal cord (64). Together, these results strongly suggest that tricaine and cooling act as anesthetics instead of simply suppressing startle and optokinetic responses via muscular paralysis.

Note that our results relate only to anesthesia in larvae and further research is needed to clarify the anesthetic effects of cooling in the adult zebrafish brain. Due to their larger size it is possible that it takes longer for the central brain to cool when compared to neurons in the spinal cord and peripheral nervous system. Crucially, while we found comparable onsets for tricaine and cooling anesthesia in larvae, a behavioral study in adults found that there are significant differences in the onset profiles between the two (18). In adults there was a delay of over 2 min between loss of equilibrium and loss of tactile response following tricaine exposure, while there was no such delay for gradual cooling.

## Conclusion

### Tricaine Is an Effective Anesthetic

The comparative effects of each treatment on the optokinetic response are summarized in Figure 7. The results of this study suggest that tricaine is an effective and potentially superior anesthetic in comparison to gradual cooling. A small number of motion-tuned neurons remains in sensory brain areas, and task-associated activity is almost completely abolished in the hindbrain. These results demonstrate that eye movements cease due to lack of behavioral drive rather than neuro-muscular paralysis, which confirms the anesthetic effect of tricaine. However, we found that even a 15-min exposure to tricaine could not completely silence motion-tuned neurons, so a higher dose of tricaine than the standard 168 mg/L concentration may be necessary to achieve higher levels of neural suppression. Of importance to the practical implementation of anesthesia, we did not observe any meaningful temporal discrepancy between the loss or recovery of reflexive eye movements and neural drive, suggesting that there is no lag between neuronal vs. behavioral onset and offset of the anesthetic effect of tricaine. Thus, observation of zebrafish movements during application of tricaine can serve as a reliable readout for the onset of anesthesia. We nonetheless caution readers that behavioral recovery took quite long (~14 min), and it is possible that full recovery of neural activity occurs before the optokinetic behavioral recovery, during time periods we did not record.

Finally, gradual cooling should not be considered an appropriate alternative anesthetic agent until the long-term effects of the calcium surge have been fully investigated.

## Data Availability Statement

The raw data supporting the conclusions of this article will be made available by the authors, without undue reservation.

## Ethics Statement

The animal study was reviewed and approved by Regierungspräsidium Tübingen in accordance with German federal law and Baden-Württemberg state law.

## Author Contributions

FDeh and AA: conceptualization. CL, FDeh, and AA: methodology and software. CL: formal analysis and data curation. CL, TB, FDeb, CS, and AA: investigation. CL and AA: writing—original draft preparation and visualization and supervision. CL, TB, FDeb, CS, FDeh, and AA: writing—review and editing. AA: project administration. FDeh and AA: funding acquisition. All authors contributed to the article and approved the submitted version.

## Funding

This work was funded by the Deutsche Forschungsgemeinschaft (DFG) grants EXC307 (CIN – Werner Reichardt Centre for Integrative Neuroscience) and INST 37/967-1 FUGG, and a Bf3R grant (No. 1328-569) from the German Federal Institute for Risk Assessment (BfR).

## Conflict of Interest

The authors declare that the research was conducted in the absence of any commercial or financial relationships that could be construed as a potential conflict of interest.

## Publisher's Note

All claims expressed in this article are solely those of the authors and do not necessarily represent those of their affiliated organizations, or those of the publisher, the editors and the reviewers. Any product that may be evaluated in this article, or claim that may be made by its manufacturer, is not guaranteed or endorsed by the publisher.
