# Supplementary material for: Efficacy of Tricaine (MS-222) and Hypothermia as Anesthetic Agents for Blocking Sensorimotor Responses in Larval Zebrafish
Source: Front Vet Sci. 2022 Mar 28;9:864573. doi: 10.3389/fvets.2022.864573 (PMC8996001; doi:10.3389/fvets.2022.864573)
Supplement: Supplementary file 1 [file Data_Sheet_1.pdf]

Supplementary Figures

Supplementary Figure 1:

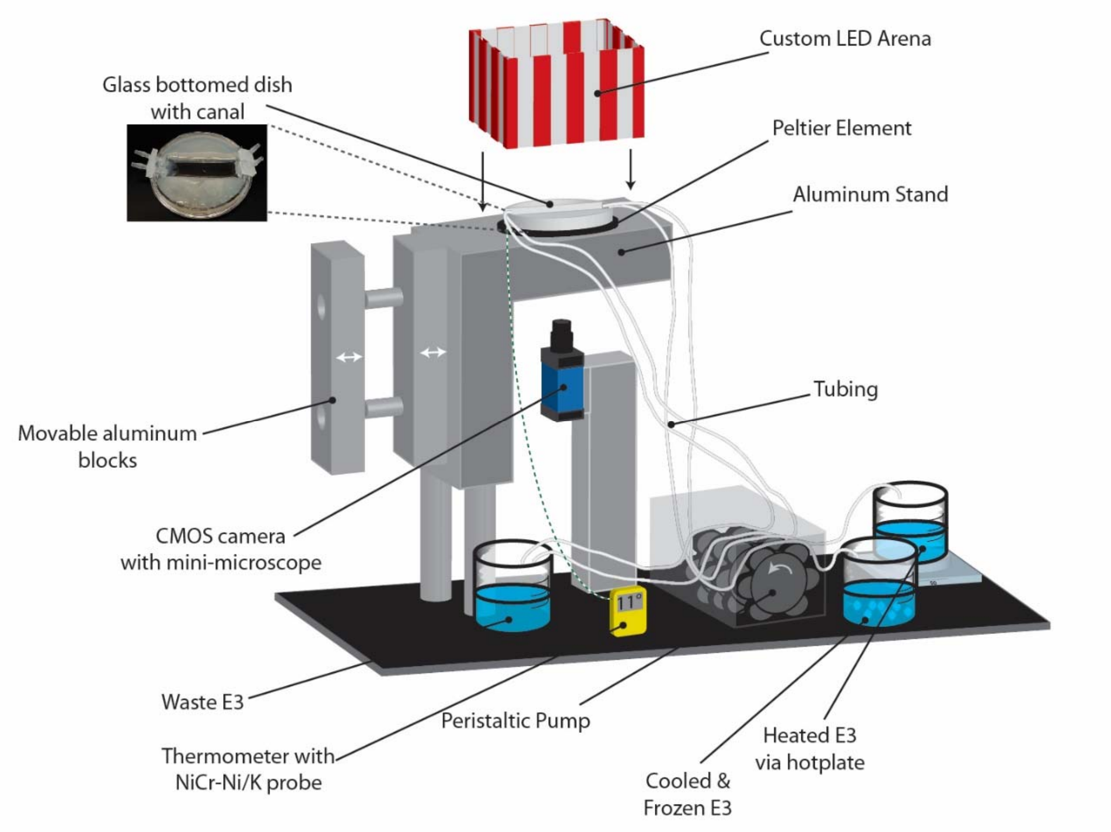

Schematic illustrating the set-up used for all behavioral experiments in immobilized fish. Movable blocks were only used for gradual cooling experiments and controls. In the case of tricaine experiments, the hot plate was removed, and the two beakers instead contained E3 containing tricaine at a defined concentration and tricaine-free E3, respectively. For control experiments, both beakers contained tricaine-free E3.

Supplementary Figure 2:

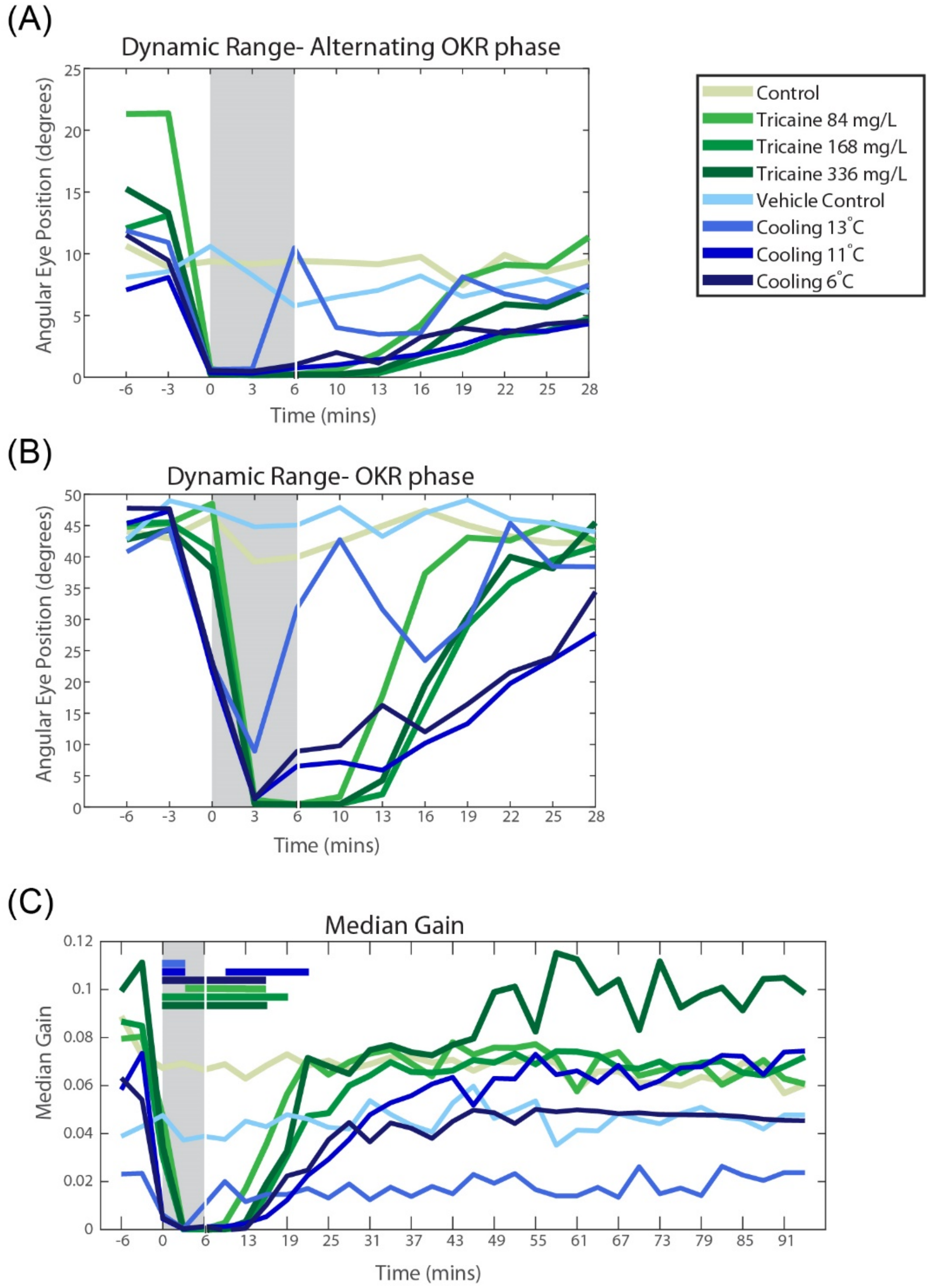

Breakdown of the dynamic range values for both the alternating optokinetic response phase (OKR) **(A)** and OKR phase **(B)** during the treatment window. The dynamic range recovered faster during the OKR phase probably due to the evoked saccades. The median gain results **(C)** were similar across conditions. In the case of tricaine, both the standard and 2x concentrations were equally effective (significant for 7 consecutive 3-minute iterations), whereas the 0.5x concentration was not effective during the first three-minute protocol iteration, but was significant for the following five 3-minute iterations. In the case of cooling, 6 degrees had the slowest recovery of the temperatures tested, and was significantly different from the 3-minute band immediately after application and the following 7* 3-minute iterations. 13 degrees was significant only for 3-minute band immediately after application and the subsequent 3-minute iteration, and fish recovered as soon as the temperature was increased. 6 degrees was also effective at the application time point and subsequent 2*3-minute iterations, lost significance for 3 minutes and then was significant for the following 2*3-minute iterations.

Supplementary Figure 3:

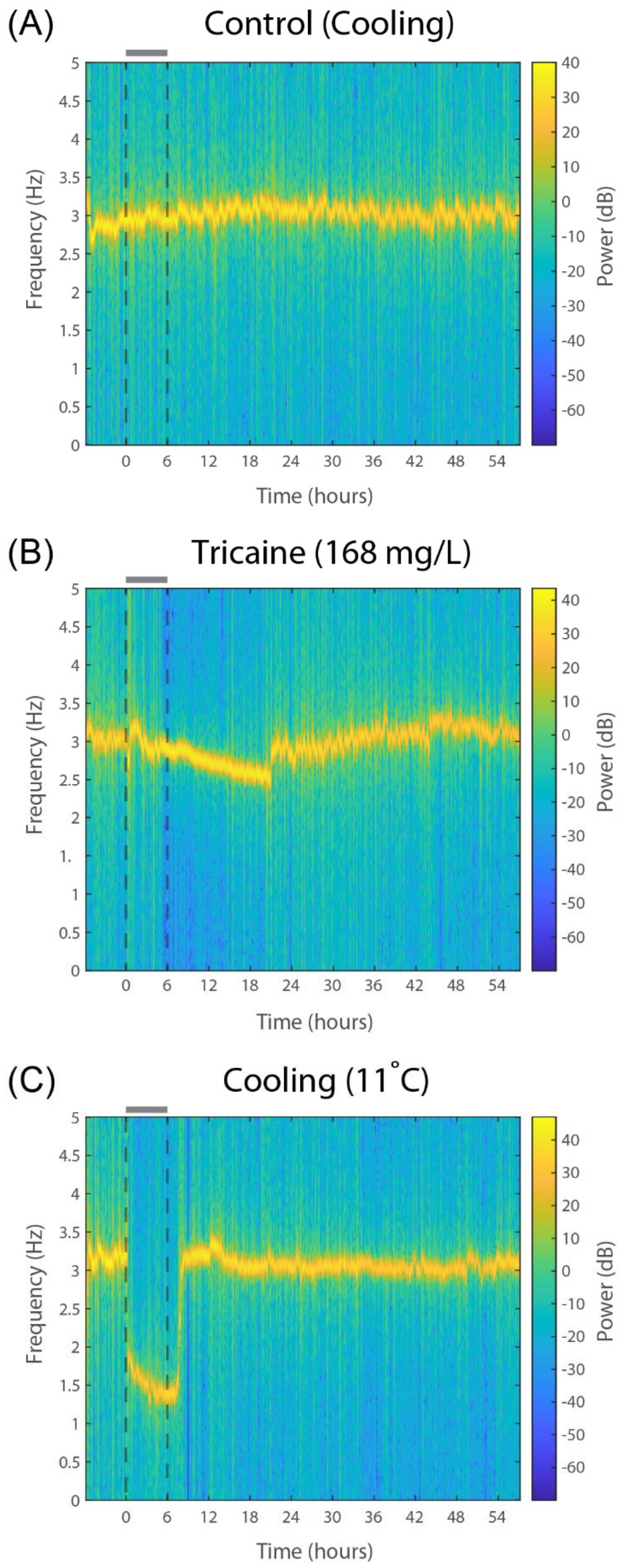

Sample of heartrate (HR) plot before, during and after application for a control larva (1,2-propanediol) **(A)**, standard tricaine treatment (168 mg/L) **(B)** and gradual cooling (11°C) **(C)**. The heatmap shows the power spectrum of the recorded heart videos. Time of exposure is indicated by the horizontal grey bars above, and vertical grey bars within the figure.

Supplementary Figure 4:

(A)

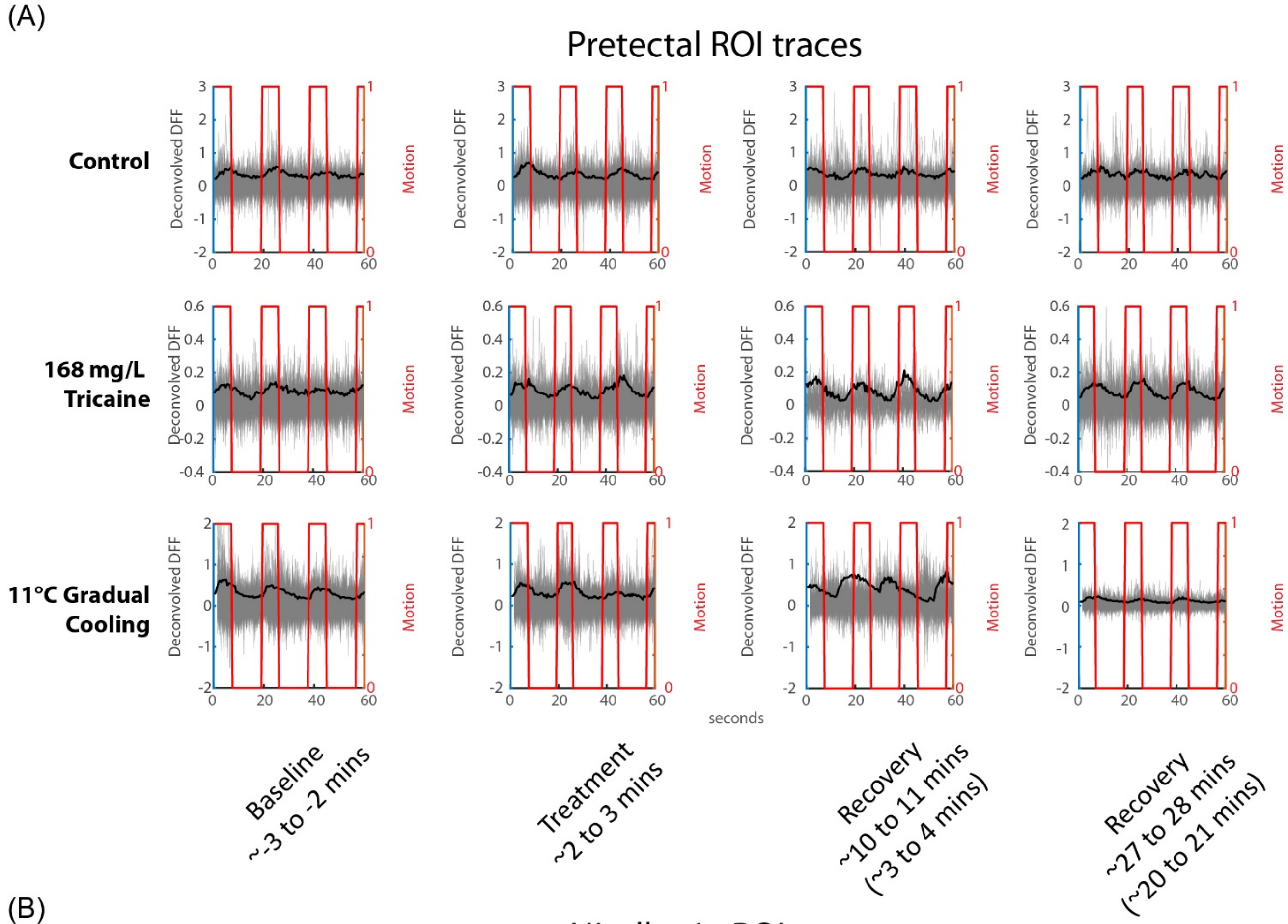

(B)

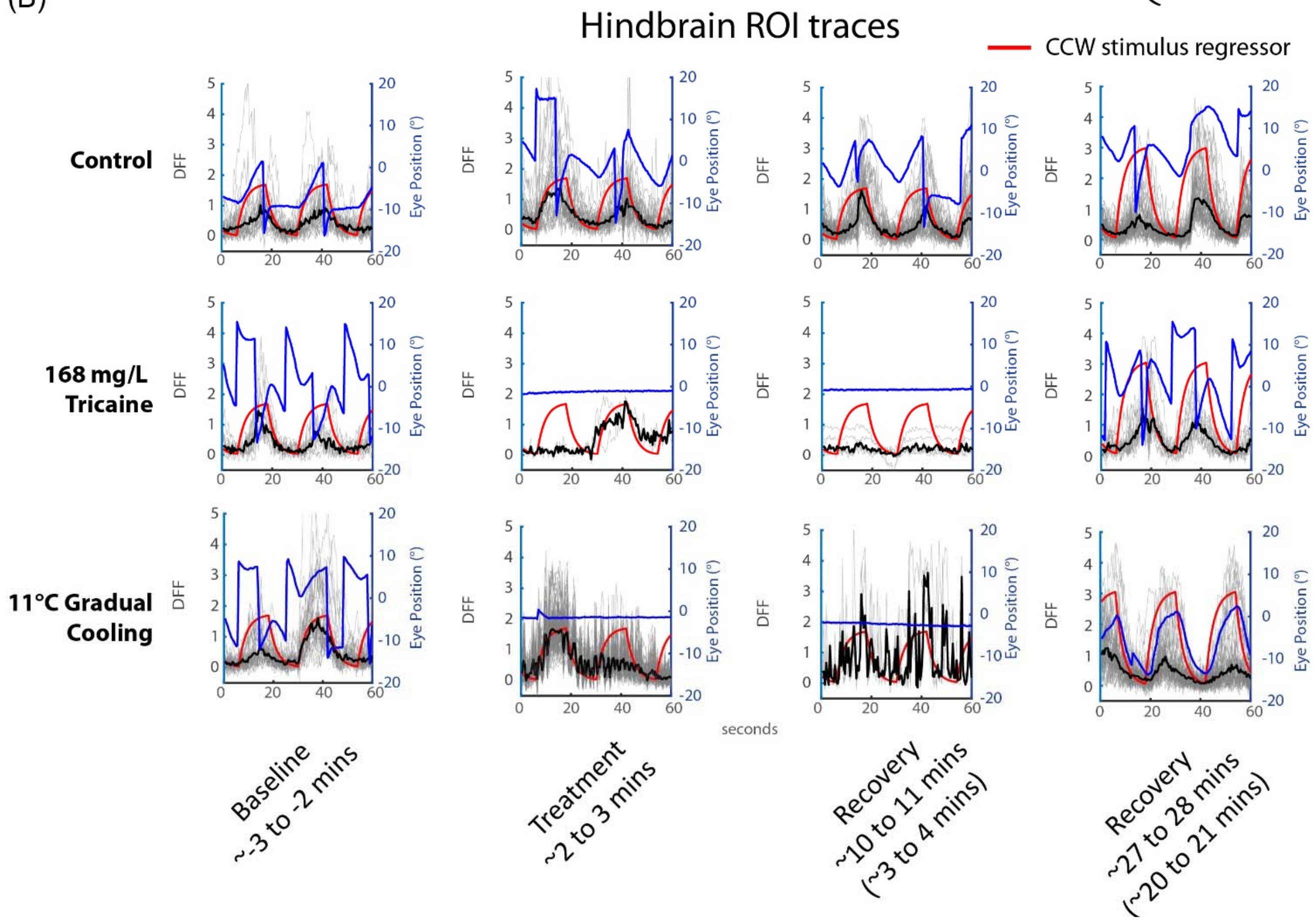

Stimulus activity of ROIs identified via automated segmentation based on stimulus regressor correlation from a sample recording of a control, 168 mg/L tricaine and 11°C gradually cooled fish. Segmented ROIs identified during individual 1-minute time bins are included (for the control larva, the plots contain 86, 74, 96 and 63 ROIs respectively, in the case of tricaine there are 100, 84, 77 and 76 ROIs, and for gradual cooling 113, 191, 123 and 97 ROIs). Note that the y-axes limits differ between experimental groups, which is likely due to differences in recording quality. The times refer to the time of anesthesia application, in the case of recovery the time since the end of anesthesia exposure is given in brackets. **(A)** Comparison of the deconvolved calcium traces used to determine the motion-sensitivity index of individual ROIs recording before during and after treatment with gradual cooling or 168 mg/L tricaine. Activity is also shown for a control experiment. In red is the motion regressor, the direction of motion alternated between clockwise (CW) and counter-clockwise (CCW). The median deconvolved fluorescence trace for all ROIs is shown in black. **(B)** Comparison of the neural activity recorded from individual cells encoding CCW eye position in a single recording before during and after treatment with gradual cooling or 168 mg/L tricaine. Activity is also shown for a control experiment. Red shows the convolved regressor trace for the CCW stimulus. Blue shows the average eye position. Each grey line represents the activity of a single neuron; the median trace is shown in black.

Supplementary Figure 5:

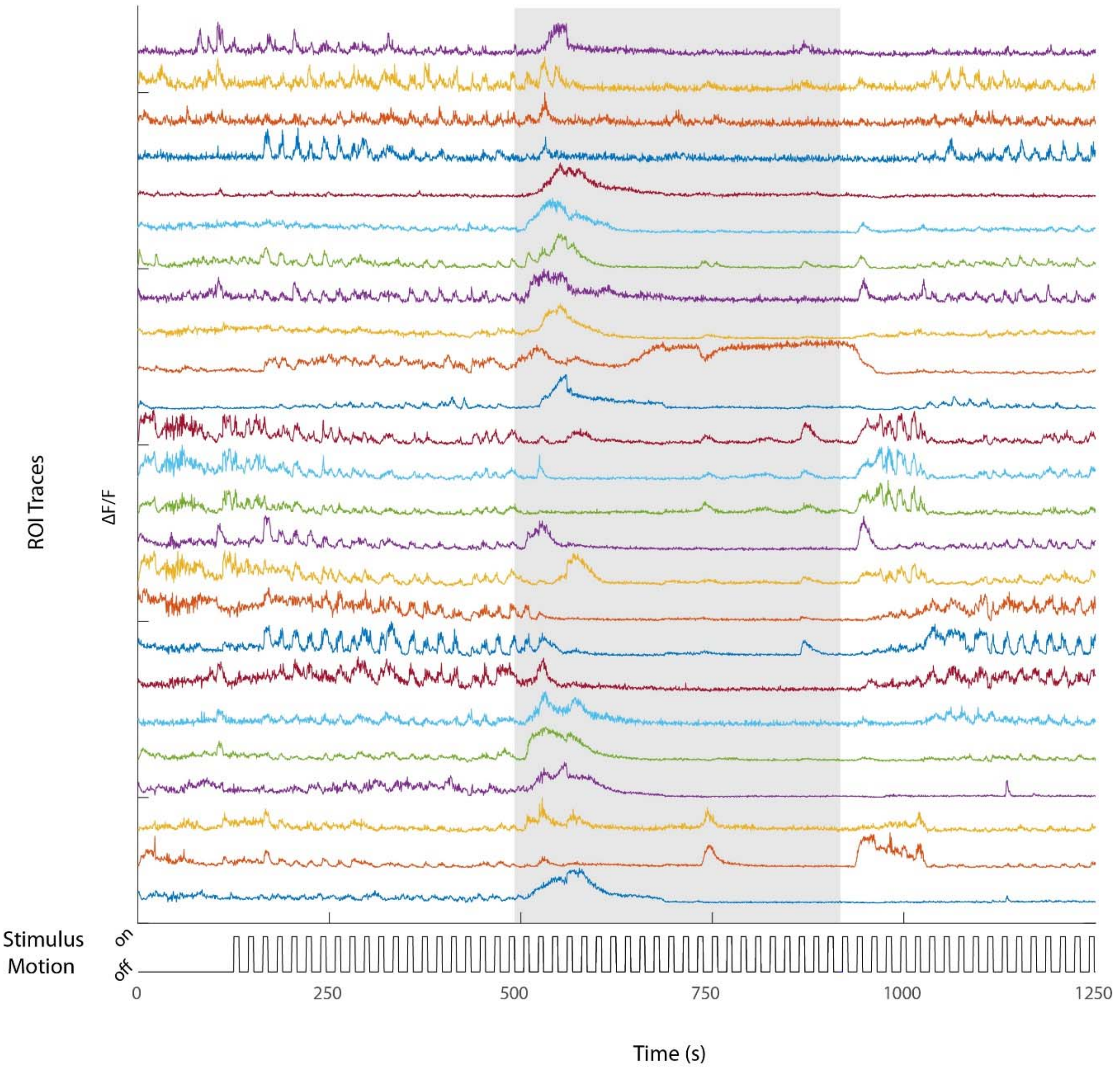

Sample traces of individual ROIs detected in a single recording carried out in the pretectum during gradual cooling treatment. Cooling was applied from 490- 910 seconds, as shown by the grey shaded area. ROI masks were automatically detected and segmented from the standard-deviation image projection of the calcium image time series via a watershed transform (Zhang and Arrenberg, 2019). This detection was independent of stimulus correlations. ROIs which were correlated to the stimulus during the baseline period with a coefficient greater than 0.3 are shown (25/371).
